# Gait Retraining for Patellofemoral Pain in Runners: An Umbrella Review of Clinical and Biomechanical Evidence

**DOI:** 10.1186/s40798-026-01051-8

**Published:** 2026-06-15

**Authors:** Ana Carolina Petri, Tamiris Beppler Martins, Taís Beppler Martins, Jaqueline de Souza, Filippo Migliorini, Nicola Maffulli, Rodrigo Okubo

**Affiliations:** 1 Departamento de Fisioterapia, Centro de Ciências da Saúde e do Esporte, Universidade do Estado de Santa Catarina, Florianópolis, SC Brazil; 2 Programa de Pós-graduação em Fisioterapia, Universidade do Estado de Santa Catarina, Florianópolis, SC Brazil; 3 Department of Trauma and Reconstructive Surgery, University Hospital of Halle, Martin-Luther University Halle-Wittenberg, Ernst-Grube-Street 40, 06097 Halle (Saale), Germany; 4 Department of Life Sciences, Health, and Health Professions, Link Campus University, Via del Casale di San Pio V, 00165 Rome, Italy; 5 Department of Orthopedics and Trauma Surgery, Eifelklinik St Brigida, Simmerath, Germany; 6 Faculty of Medicine and Psychology, University “La Sapienza” of Rome, Rome, Italy; 7 School of Pharmacy and Bioengineering, Keele University Faculty of Medicine, Stoke On Trent, ST4 7QB UK; 8 Centre for Sports and Exercise Medicine, Barts and the London School of Medicine and Dentistry, Mile End Hospital, Queen Mary University of London, London, E1 4DG UK; 9 Departamento de Fisioterapia, Centro de Ciências, Tecnologias e Saúde, Universidade Federal de Santa Catarina, Araranguá, Brazil

**Keywords:** Gait retraining, Patellofemoral pain, Biomechanics, Injury prevention, Sports physiotherapy

## Abstract

**Background:**

Gait retraining has been proposed as a strategy to modify running biomechanics and reduce symptoms in runners with patellofemoral pain (PFP). However, review-level evidence remains heterogeneous regarding clinical effects, biomechanical mechanisms, and preventive applicability.

**Objectives:**

To critically evaluate the methodological quality and synthesise review-level evidence on gait retraining for the management of patellofemoral pain (PFP) in runners while secondarily summarising preventive findings when available.

**Methods:**

Umbrella review conducted in accordance with Joanna Briggs Institute guidelines and reported according to PRISMA 2020. PubMed/MEDLINE and Scopus were searched from inception to March 2026, complemented by manual searches. Systematic reviews (with or without meta-analysis) and structured narrative reviews investigating gait retraining in runners were included. Clinical conclusions were prioritised from reviews focused on symptomatic runners with patellofemoral pain, whereas broader biomechanical or preventive evidence was interpreted contextually. Methodological quality was assessed using AMSTAR-2 for systematic reviews and SANRA for narrative reviews.

**Results:**

Eight reviews were included: five systematic reviews and three narrative or mixed-methods reviews. AMSTAR-2 rated four systematic reviews as high quality and one as low quality, while SANRA rated two narrative or mixed-methods as high quality and one as good quality. Gait retraining strategies primarily involved increasing cadence by 5–10%, reducing vertical impact through soft-landing cues, modifying foot-strike pattern, and auditory or visual biofeedback, usually delivered progressively with gradual feedback withdrawal. The most consistent findings related to short-term improvements in pain and function and reductions in selected loading-related biomechanical variables, particularly with cadence-based and soft-landing strategies. Findings for injury prevention, foot-strike modification, and longer-term outcomes were less consistent.

**Conclusions:**

Gait retraining may be a feasible adjunctive intervention for runners with patellofemoral pain, particularly when focused on modest cadence increases and/or softer-landing cues within supervised, progressively faded-feedback protocols. However, heterogeneity across protocols and the predominance of short-term outcomes limit stronger conclusions. Preventive effects remain uncertain, and high-quality controlled longitudinal studies are needed to clarify long-term clinical relevance and identify runners most likely to benefit.

**Registration:**

PROSPERO (CRD420251145069).

**Supplementary Information:**

The online version contains supplementary material available at 10.1186/s40798-026-01051-8.

## Key Points

- Simple running-style changes may reduce pain and improve function in runners with patellofemoral pain.
- A slightly higher step rate and softer landing may reduce stress on the kneecap.
- Long-term and prevention effects remain unclear.

**Supplementary Information:**

The online version contains supplementary material available at 10.1186/s40798-026-01051-8.

## Introduction

Running is one of the most accessible and widely practised physical activities worldwide, offering substantial cardiovascular, metabolic, and mental health benefits when performed regularly [1–3]. However, these benefits are accompanied by a high incidence of musculoskeletal injuries, particularly in the lower limbs, and especially with the knee [2, 3]. Among knee conditions affecting runners, patellofemoral pain (PFP) is frequent and is often associated with repetitive overload and biomechanical alterations that negatively impact performance and sustained participation in the sport [1, 4].

PFP is insidious, commonly described as pain around or behind the patella, and is aggravated during activities that increase patellofemoral joint loading, such as squatting, stair climbing, running, and jumping [5]. Although its aetiology is multifactorial, biomechanical factors, such as increased hip adduction and internal rotation, elevated vertical loading rates, and altered step mechanics have been proposed to contribute to patellofemoral joint loading; however, these variables should be interpreted within a broader multifactorial framework rather than as isolated causal determinants of patellofemoral pain [4, 6, 7].

In this context, gait retraining, grounded in principles of motor learning, has emerged as a promising and potentially effective strategy to reduce joint stress and optimise running mechanics. Common approaches include increasing cadence, modifying foot-strike patterns, and adjusting trunk or pelvic position [8, 9]. Although promising, the current body of evidence remains methodologically heterogeneous, and conclusions on its efficacy and clinical applicability vary [1, 10].

Recent systematic and narrative reviews differ in their populations, protocols, and outcomes, complicating the consolidation of evidence on gait retraining for PFP [1, 6, 11–13]. Nonetheless, increasing cadence may reduce hip adduction, vertical loading rates, and ground reaction forces, thereby lowering patellofemoral stress and improving function [1, 6]. Strategies that leverage biofeedback and real-time visual or auditory cues have also demonstrated the potential to promote lasting movement adaptations and reduce pain in runners with PFP [11, 14, 15]. However, substantial gaps remain regarding protocol standardisation and the generalizability of findings across recreational and competitive runners.

Given this scenario, the aim of this umbrella review is to critically synthesise systematic and structured narrative reviews that examine gait retraining for the management of patellofemoral pain in runners while considering preventive evidence as a secondary outcome. To our knowledge, no previous umbrella review has specifically summarised the body of review-level evidence on this topic. This review seeks to identify key biomechanical mechanisms, evaluate effects on pain and function, and discuss clinical implications for physiotherapy practice. We hypothesise that gait retraining would be associated with reductions in pain and in biomechanical variables proposed to influence patellofemoral joint loading, whereas evidence for prevention and long-term sustainability would remain uncertain.

## Methods

### Study Design

This study is characterised as an umbrella review, that is, a review of systematic reviews with a restricted thematic scope. An umbrella approach was chosen, because the available literature includes multiple reviews with partially overlapping questions, as well as clinically oriented reviews that discuss implementation and mechanisms. The study was registered in the PROSPERO database for systematic reviews (CRD420251145069).

The review was conducted in accordance with the Joanna Briggs Institute (JBI) methodological guidelines for umbrella reviews [16] and adhered to the PRISMA 2020 (Preferred Reporting Items for Systematic Reviews and Meta-Analyses) recommendations.

### Guiding Question

The research question was structured according to the PICO framework: P (Population)—symptomatic recreational or competitive runners with patellofemoral pain; broader running populations were considered only when reviews reported preventive outcomes or mechanistic biomechanical findings relevant to PFP separately; I (Intervention)—gait retraining strategies, including cadence adjustment, foot-strike modification, biofeedback, postural instruction, or combinations of these approaches; C (Comparator)—control groups receiving no intervention or alternative interventions; and O (Outcomes)—pain, function, biomechanical parameters (kinematic and kinetic), safety/adverse events, and incidence or recurrence of patellofemoral pain (PFP). Based on this framework, the guiding research question was: “What evidence from systematic reviews supports the efficacy, safety, and clinical applicability of gait retraining for the management of patellofemoral pain in runners, and what preventive findings are available as secondary evidence?".

### Information Sources and Search Strategy

Electronic searches were conducted in PubMed/MEDLINE and Scopus, covering the period from database inception through March 2026. These databases were selected, because they provide broad coverage of sports medicine, rehabilitation, and biomedical literature, and Scopus indexes journals not always captured in PubMed/MEDLINE. The search strategy combined descriptors and free-text terms with Boolean operators, following the structure: (“running” OR “runners” OR “running-related injuries” OR “patellofemoral pain” OR “PFP” OR “anterior knee pain”) AND (“gait retraining” OR “running technique” OR “running cadence” OR “foot strike” OR “step rate” OR “stride length”) AND (“systematic review” OR “meta-analysis” OR “review”). A manual search was also performed in the reference lists of the included reviews and in preprint repositories (medRxiv, SportRxiv) to identify relevant publications not yet indexed.

### Eligibility Criteria

Eligible studies included systematic reviews (with or without meta-analysis) and high-quality narrative, mixed-methods, or clinically appraised reviews that investigated gait retraining strategies in recreational or competitive runners. For the primary synthesis, preference was given to reviews focused on symptomatic runners with PFP; reviews including asymptomatic or mixed populations were retained only when biomechanical findings relevant to PFP were clearly identifiable and were interpreted as contextual evidence rather than direct clinical evidence. Reviews had to report outcomes related to pain, function, biomechanical parameters, safety/adverse events, or knee-related symptoms; explicitly describe their search strategies and eligibility criteria; and provide an appraisal of the included primary studies or clearly describe their methodological approach. Exclusion criteria comprised editorials, theoretical essays, opinion papers, integrative reviews not focused on gait retraining, and reviews examining isolated strengthening, footwear interventions, insoles, or orthotic devices. Reviews were classified as non-systematic when they lacked a reproducible search strategy, explicit eligibility criteria, or a clear description of how evidence was selected and synthesised. Because gait retraining is an emerging field with relatively few review-level syntheses, structured narrative reviews were considered only when they focused on empirical studies, reported minimum methodological transparency, and achieved a SANRA score > = 10/12. Their findings were used mainly to contextualise mechanisms, delivery strategies, and implementation issues, whereas conclusions on clinical effectiveness were prioritised according to review design, methodological quality, and consistency of findings.

### Data Extraction and Synthesis

Screening of titles, abstracts, and full texts was conducted independently by two reviewers (authors of this study) using the Rayyan^®^ platform (Qatar Computing Research Institute, Doha, Qatar). Rayyan enabled the application of predefined inclusion/exclusion criteria, tracking of decisions, and resolution of conflicts by consensus. A third reviewer was consulted when necessary. The selection process adhered to PRISMA 2020 guidelines, documenting the number of records identified, screened, included, and excluded, along with the reasons for exclusion. Outcome data were extracted according to intervention type, comparator when available, outcome domain (pain, function, biomechanics, safety, and prevention), and timepoint. Follow-up was categorised as immediate, short-term (> 0–3 months), or mid-term (> 3–6 months) whenever reported.

### Methodological Quality Assessment

The methodological quality of systematic reviews was assessed independently by two reviewers using the AMSTAR-2 tool (A MeaSurement Tool to Assess Systematic Reviews, version 2) [17], recommended for reviews, including randomised clinical trials and observational studies. For narrative or applied reviews, the SANRA scale (Scale for the Assessment of Narrative Review Articles) [18] was used to evaluate justification, coherence, methodology, critical discussion, and reference adequacy. To reduce subjectivity, a SANRA score > = 10/12 was required for inclusion. No formal certainty-of-evidence framework, such as GRADE, was applied. Therefore, the synthesis avoids formal certainty descriptors and instead describes the consistency, direction, and methodological limitations of findings across included reviews. This combined methodological approach ensured an appropriate appraisal of reviews with different designs, thereby enhancing comparability, transparency, and the robustness of evidence interpretation.

### Evidence Synthesis and Overlap Analysis

To reduce duplication of evidence derived from the same primary studies, overlap between the included reviews was assessed [19]. An overlap matrix was constructed to identify repeated studies and estimate the degree of redundancy. Overlap was incorporated into the interpretation by giving greater weight to findings replicated across higher-quality reviews while avoiding repeated counting of the same primary studies as independent support.

### Methodological Rigour and Transparency

All stages, search, screening, data extraction, and quality assessment, were performed independently by pairs of reviewers. Discrepancies were resolved by consensus and documented. The protocol for this umbrella review was developed a priori, in accordance with JBI recommendations, and a summary of the protocol and database-specific search strategy is provided in the Supplementary Material.

## Results

### Study Selection

**Fig. 1:**
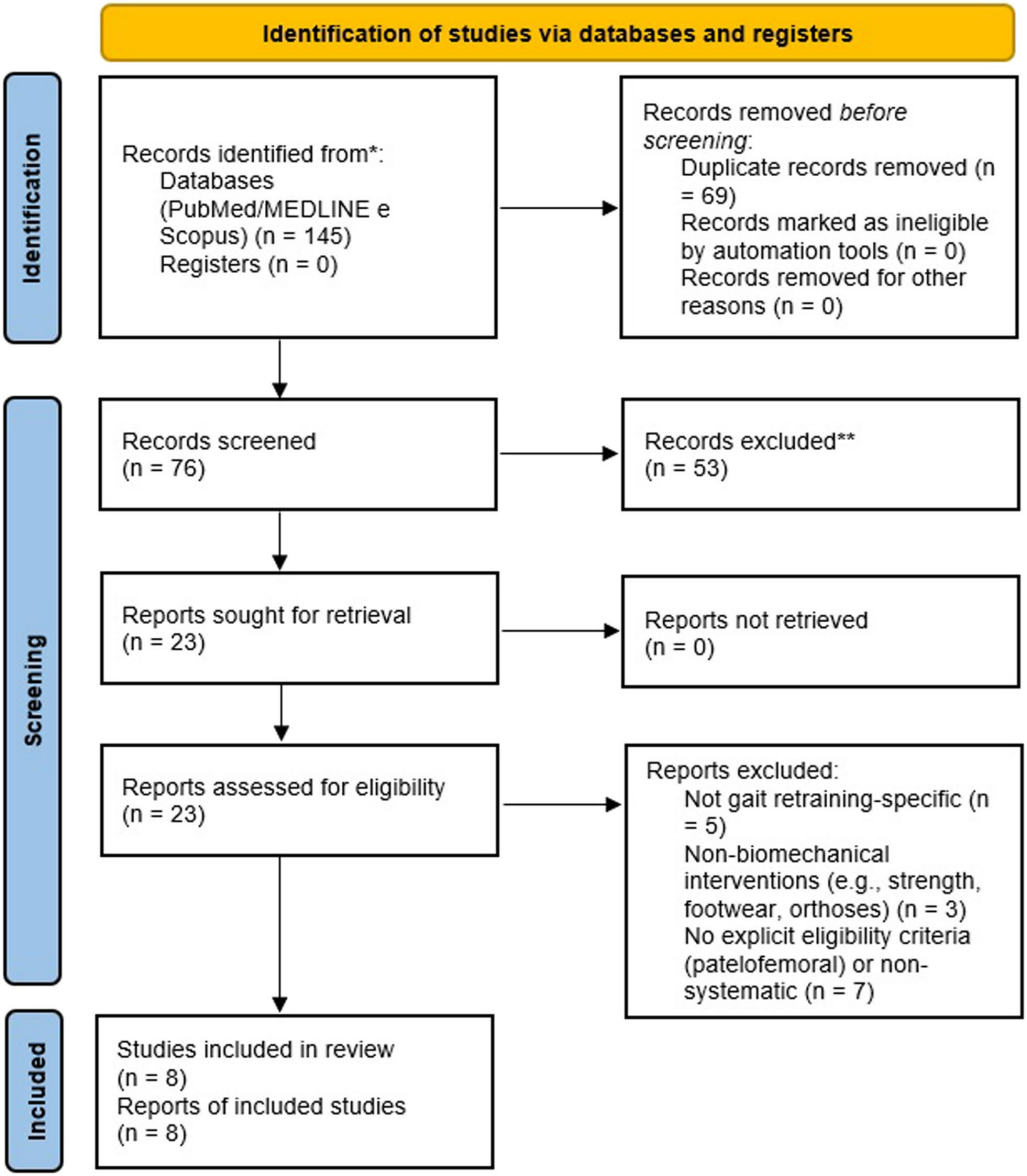
PRISMA 2020 flow diagram adapted for the umbrella review on gait retraining for the management of patellofemoral pain in runners, with preventive evidence considered as secondary evidence

The initial search across PubMed/MEDLINE and Scopus yielded 145 potentially relevant records. After removing 69 duplicates, 76 records were screened by title and abstract using the Rayyan^®^ platform. Twenty-three articles were selected for full-text assessment, of which eight reviews met all eligibility criteria and were included in the final synthesis. Five studies were classified as systematic reviews, with or without meta-analysis [1, 6, 11, 12, 20]. Three studies were classified as narrative or mixed-methods reviews [13–15]. The main reasons for excluding full-text articles were the absence of specific analysis of gait retraining (*n* = 5); a focus on non-motor or non-biomechanical interventions, such as isolated strengthening, footwear, or orthoses (*n* = 3); and the lack of explicit eligibility criteria, reproducible search methods, or a clear synthesis process (*n* = 7). A detailed PRISMA 2020 flow diagram is presented in Fig. 1, and the general characteristics of the included reviews, including population, gait retraining interventions, outcomes, and key conclusions, are summarised in Table 1.

**Table 1:** General characteristics of the included reviews on gait retraining for patellofemoral pain in runners

Author (year)	Type of review	Population/included studies	Gait retraining interventions	Outcomes	Conclusions
Agresta et al. (2015) [11]	Systematic review	10 studies; healthy runners, PFP, and CECS	Real-time augmented feedback (visual, auditory); cadence increase; stride-length adjustment; foot-strike modification; faded-feedback protocols	Impact forces (VIP, VALR, VILR); hip/pelvis kinematics; step rate/length; pain (PFP)	Augmented feedback reduces impact variables and improves kinematics; some symptom improvement in PFP; protocols heterogeneous; evidence preliminary
Neal et al. (2016) [12]	Systematic review + meta-analysis	28 studies, runners with PFP	Visual biofeedback; increased cadence; softer-landing cues; impact reduction strategies; retraining protocols delivered during treadmill running	Pain and function; hip/knee kinematics; impact variables; step rate; loading measures	Consistent short-term improvements in pain, function, and lower-limb kinematics; gait retraining effective in reducing hip adduction and dynamic valgus; long-term effects remain uncertain
Alexander et al. (2022) [1]	Systematic review + meta-analysis (RCTs)	30 trials, novice and recreational runners	“Land softer” cue; cadence increase; impact-reduction strategies; supervised treadmill-based retraining	Running-related knee injury incidence; PFP symptoms; vertical loading rates; step rate	One preventive trial suggested that a soft-landing cue may reduce knee injury risk (~ 66%); however, preventive findings were not derived from runners with established PFP and should be interpreted cautiously; small improvements in PFP symptoms; other preventive strategies show limited or inconsistent effects
Anderson et al. (2022) [6]	Systematic review + meta-analysis	37 studies; injured runners (including PFP)	Cadence increase (5–10%); step-rate modification; impact-reduction cues; real-time feedback during treadmill running	Patellofemoral joint force; knee extensor moment; vertical loading rates; lower-limb kinematics	Increasing cadence consistently reduces PFJ loading and knee extensor moment; evidence on injury risk reduction or performance effects is lacking
Figueiredo et al. (2025) [20]	Systematic review	18 studies; recreational and trained runners	Cadence increase (5–10%); stride-length reduction; impact-reduction cues; supervised treadmill retraining	Vertical loading rates; impact forces; stride length; oscillation; hip/knee kinematics; running economy	Increasing cadence reduces impact variables and improves lower-limb alignment without compromising running economy; potential preventive effects for PFP and tibial stress injuries
Barton et al. (2016) [13]	Mixed-methods review (systematic evidence synthesis + expert interviews)	46 studies (primarily asymptomatic runners; 5 injured populations; 4 studies with clinical outcomes)	Step-rate increase; transition from rearfoot to mid/forefoot strike; cues to reduce hip adduction; strategies to reduce impact loading; modifications of step width and proximal kinematics	Clinical outcomes (pain, function, compartment pressure) and biomechanical variables (impact loading, hip adduction, pelvic drop, strike pattern, step rate, step width, proximal kinematics)	Clinical findings were sparse but suggested possible benefits of running retraining for PFP and anterior exertional lower-leg pain; substantial biomechanical evidence and expert opinion support tailored, diagnosis-specific approaches
Fyock et al. (2020) [14]	Critically Appraised Topic (CAT)	3 studies; adult recreational runners with PFP	Real-time visual feedback (mirror or computer display) + verbal cues; 8 sessions over 2 weeks; feedback increased first 4 sessions and faded over last 4	Pain; function (LEFS/LEFI); hip adduction; pelvic drop; hip internal rotation; knee abduction mechanics	Consistent short- and long-term pain reduction and improved lower-limb kinematics following visual-feedback gait retraining; level 2 evidence supports its use for PFP
Gaudette et al. (2022) [15]	Narrative review	Multiple gait-retraining studies summarized; injured runners (primarily PFP and CECS); includes both symptomatic and asymptomatic runners in biomechanical sections	Cadence increase; hip adduction reduction; forward trunk lean; foot-strike modification (mid/forefoot); tibial-acceleration feedback; faded-feedback programs; treadmill and overground retraining	Pain and function in PFP; hip adduction; pelvic drop; trunk flexion; loading rates (VIP, VALR, VILR); tibial acceleration; joint moments; step rate and footstrike angle	Gait retraining can improve biomechanics and reduce pain in PFP and CECS; evidence for other RRIs is limited; individualized, diagnosis-specific approaches and faded-feedback designs are recommended; stronger RCT evidence is needed

VIP = vertical impact peak; VALR = vertical average loading rate; VILR = vertical instantaneous loading rate; PFJ = patellofemoral joint; PFP = patellofemoral pain; CECS = chronic exertional compartment syndrome; RCT = randomized controlled trial; CAT = critically appraised topic; LEFS = Lower Extremity Functional Scale; LEFI = Lower Extremity Functional Index; VAS = visual analogue scale; HADD = hip adduction angle; HABD = hip abduction angle; HABDM = hip abduction moment; HIR = hip internal rotation; CPD = contralateral pelvic drop; KABD = knee abduction angle; KFL = knee flexion; RRIs = running-related injuries; SD = standard deviation

### Methodological Quality Assessment of the Included Reviews

#### Systematic Reviews (AMSTAR-2)

Using the AMSTAR-2 tool, four systematic reviews were rated as high methodological quality [1, 6, 12, 20], whereas one systematic review was rated as low methodological quality [11]. In one systematic review rated as low quality [11], several methodological limitations were identified, including the absence of a preregistered protocol, lack of risk-of-bias assessment, and no excluded-study list [11]. Strengths of high-quality reviews included comprehensive searches, duplicate screening and extraction, and use of standardised risk-of-bias tools. Among these high-quality systematic reviews, remaining limitations mainly involved incomplete reporting of funding sources for primary studies and inconsistent assessment of publication bias. The methodological quality of the systematic reviews, assessed using the AMSTAR-2 tool, is presented in Table 2.

**Table 2:** Methodological quality assessment of systematic reviews according to AMSTAR-2

Domain/Item	Agresta et al. (2015) [11]	Neal et al. (2016) [12]	Alexander et al. (2022) [1]	Anderson et al. (2022) [6]	Figueiredo et al. (2025) [20]
1 Clearly defined PICO question	✓	✓	✓	✓	✓
2 Protocol registered before the review	–	(✓)	✓	✓	✓
3 Justification for the included study designs	(✓)	✓	✓	✓	✓
4 Comprehensive database search	(✓)	✓	✓	✓	✓
5 Study selection performed in duplicate	(✓)	✓	✓	✓	✓
6 Data extraction performed in duplicate	(✓)	✓	✓	✓	✓
7 List of excluded studies with justification	–	✓	✓	✓	✓
8 Detailed description of included studies	(✓)	✓	✓	✓	✓
9 Risk-of-bias assessment of individual studies	–	✓	✓	✓	✓
10 Reporting of funding sources for primary studies	–	(✓)	(✓)	(✓)	✓
11 Appropriate methods for meta-analysis	–	✓	✓	✓	✓
12 Consideration of RoB impact on results	–	✓	✓	✓	✓
13 Interpretation of results considering RoB	(✓)	✓	✓	✓	✓
14 Discussion of heterogeneity	(✓)	✓	✓	✓	✓
15 Assessment of publication bias	–	(✓)	✓	✓	✓
16 Declaration of conflicts of interest and funding	(✓)	✓	✓	✓	✓
Final AMSTAR-2 rating	Low	High	High	High	High

✓ = fully met; (✓) = partially met; – = not met

**Fig. 2:**
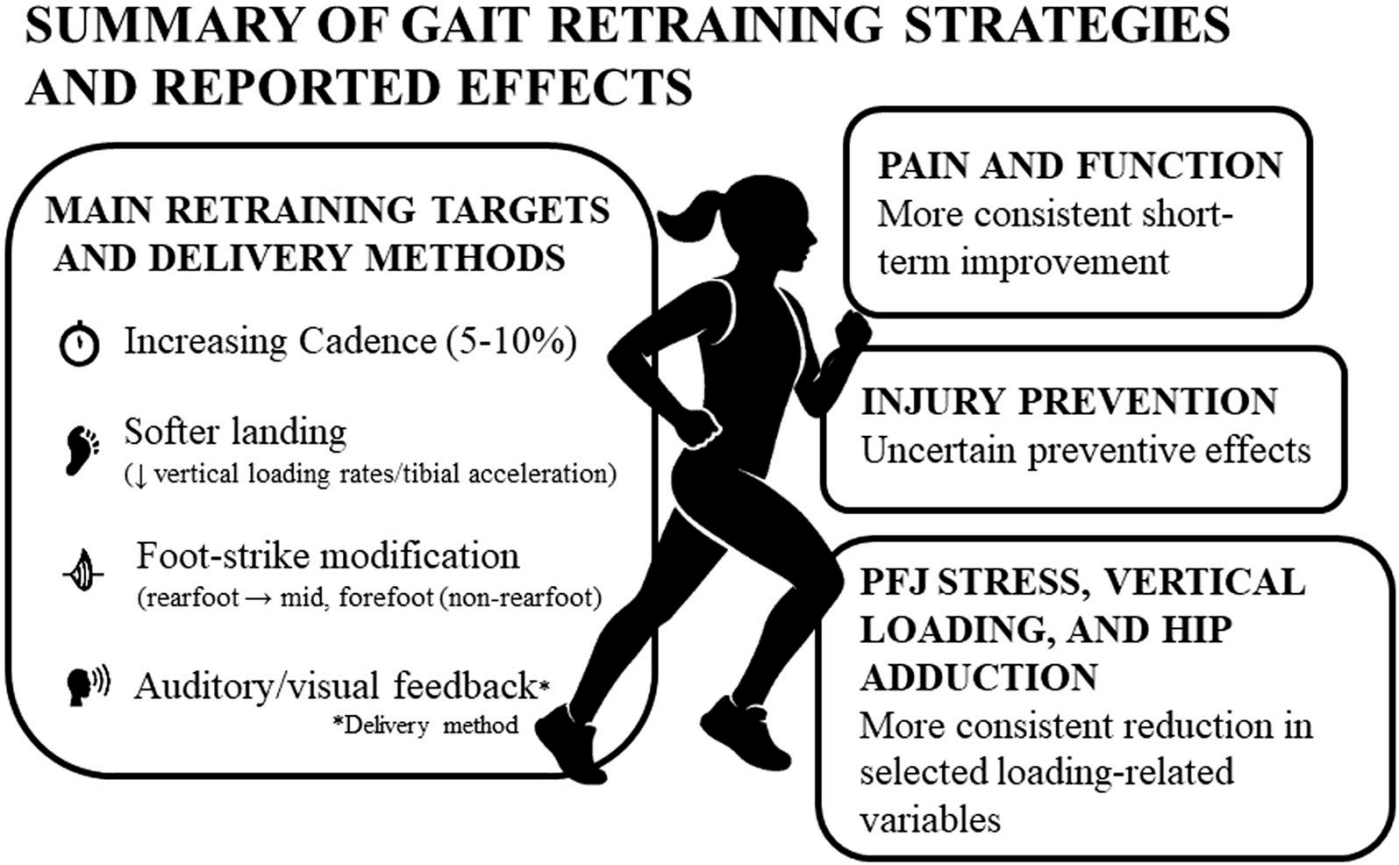
Summary of gait retraining strategies and reported biomechanical and clinical effects in runners with patellofemoral pain. Cadence increase and softer-landing cues are presented as the main retraining targets, while auditory or visual feedback is presented as a delivery method used to facilitate these changes and to target variables, such as hip adduction, trunk lean, or foot-strike modification. Foot-strike modification refers to transitions from a rearfoot to a midfoot or forefoot strike pattern. The figure summarises outcomes that were more consistently reported across reviews, including short-term improvements in pain and function and reductions in selected loading-related biomechanical variables, as well as the uncertainty surrounding injury prevention findings

#### Narrative Reviews (SANRA)

The narrative reviews appraised with the SANRA scale are summarised in Table 3. All three reviews [13–15] demonstrated good to high methodological quality, with strengths in clarity of aims, strong referencing, and coherent clinical reasoning. Limitations primarily involved incomplete reporting of search strategies.

**Table 3:** Methodological quality assessment of narrative reviews according to SANRA

SANRA Item	Gaudette et al. [15]	Fyock et al. [14]	Barton et al. [13]
1. Justification of the importance of the topic	2	2	2
2. Clarity of aims or research questions	2	2	2
3. Description of the literature search	1	1	1
4. Referencing of key statements	2	2	2
5. Scientific reasoning/coherence of argumentation	2	2	2
6. Appropriate presentation of data	2	1	2
Total score (0–12)	11/12	10/12	11/12
Overall rating	High quality	Good quality	High quality

#### Overlap Analysis

The overlap analysis yielded a corrected covered area (CCA) of 0.18 (< 25%), indicating low overlap among primary studies and suggesting that each review contributed largely unique evidence [19]. The greatest overlap was observed among reviews addressing clinical trials focused on cadence manipulation and visual biofeedback [12, 13, 20]. The low degree of overlap was considered during synthesis to reduce redundancy and avoid overweighting repeated primary studies.

#### Narrative Synthesis of the Findings

Cadence-based retraining, typically compared with habitual running or no retraining, was the strategy with the most consistent findings across the included reviews. Convergent biomechanical findings were mainly observed for cadence increases of approximately 5–10% and soft-landing cues, which were commonly associated with reductions in selected loading-related variables, including patellofemoral joint loading proxies, vertical loading rates, and impact-related measures when these outcomes were reported [1, 6, 12, 20]. These effects appeared more consistent for impact-loading variables such as VALR and/or IVLR than for proximal kinematic variables. Across reviews focused on symptomatic runners with PFP, short-term improvements were more consistently reported for pain during running and self-reported function than for usual pain at rest or broader symptom measures. However, the magnitude and durability of these clinical changes varied across reviews, and mid-term follow-up data remained sparse. Therefore, the available evidence suggests convergence for short-term symptom-related outcomes but does not establish consistent long-term clinical effects.

Findings were less consistent for foot-strike modification and proximal kinematic outcomes, such as hip adduction, pelvic drop, and knee valgus. Foot-strike modification was often delivered together with cadence or impact-reduction cues, making it difficult to isolate its independent contribution. Similarly, auditory and visual feedback were better interpreted as delivery methods for retraining strategies rather than as independent interventions. Visual feedback was commonly used to target hip adduction, dynamic knee valgus, trunk lean, or softer-landing mechanics; however, not all kinematic variables changed consistently across studies, and some proximal measures showed mixed or null findings [13–15].

Regarding prevention, one systematic review reported a reduced risk of running-related knee injury with a “land softer” cue; however, this finding was derived from preventive cohorts rather than runners with established PFP and should, therefore, be interpreted cautiously [1]. Other preventive or combined programmes showed inconsistent effects. Safety was reported infrequently; among reviews that described adverse events or tolerability, gait retraining appeared generally well-tolerated when introduced gradually, although symptom aggravation or distal calf/Achilles discomfort was reported when biomechanical changes were introduced too rapidly [12, 14]. A summary of the main gait retraining strategies, their reported biomechanical and clinical effects, and the remaining uncertainty regarding injury prevention is presented in Figure 2.

## Discussion

This umbrella review synthesised evidence from five systematic reviews and three narrative or mixed-methods reviews, providing an overview of gait retraining as a clinical strategy to manage patellofemoral pain (PFP) in runners. Across review designs, the most consistent findings support cadence increases of approximately 5–10% and softer-landing cues strategies that can reduce variables proposed to influence patellofemoral joint loading. However, because the evidence base is heterogeneous and partly supported by structured narrative reviews, gait retraining should be interpreted as a potentially useful component, rather than a stand-alone central component, of rehabilitation for PFP. Although a small number of narrative or clinically appraised reviews were included to address implementation details, feedback delivery, and mechanistic interpretation not consistently covered by systematic reviews, this choice may have introduced additional risk of bias and may have influenced the coherence of the synthesis. Therefore, clinical implications should be interpreted cautiously and primarily in light of findings that converge across systematic reviews.

### Mechanistic Consistency: Cadence, Foot-Strike Pattern, and Loading

Increasing running cadence is the most consistently supported strategy. Cadence increments of 5–10% reduce peak knee flexion, knee extensor moment, hip adduction, and vertical ground reaction forces, biomechanical factors proposed to contribute to elevated patellofemoral joint loading [6, 12, 21]. Narrative reviews and applied evidence [13–15] corroborate these findings, indicating that these adjustments were reported alongside short-term symptomatic relief, although direct causal pathways remain uncertain. Although many PFP interventions involve simultaneous adjustments in foot-strike pattern, transitioning from a rearfoot to a midfoot or forefoot strike appears to play a secondary, yet supportive, role. Studies indicate decreases in impact peaks and loading rates, although results are more variable than those from cadence-focused interventions [22, 23]. Broader evidence on foot-strike modification suggests that transitioning from a rearfoot to a non-rearfoot pattern may reduce vertical loading rates and patellofemoral joint stress, but may also increase distal loading at the ankle–Achilles complex, supporting a cautious and individualised implementation of this strategy [24]. The interaction between stride frequency and strike pattern likely reflects a coordinated motor adaptation, and retention of these changes has been observed in short-to-mid-term follow-up [2, 25].

### Clinical Outcomes: Pain, Function, and Injury Risk

Across systematic reviews, gait retraining was generally associated with short-term reductions in pain and improvements in function among individuals with PFP [12, 20]. Although some reviews reported clinical improvements alongside changes in lower-limb kinematics or loading-related variables, the available evidence does not establish that these biomechanical changes directly caused symptom improvement. Therefore, biomechanical adaptations should be interpreted as plausible explanatory factors rather than confirmed mediators of clinical response. Pain and function in PFP are multifactorial, and reductions in joint loading do not necessarily translate into symptom improvement in all individuals [6, 7, 26]. Most clinical trials assessed outcomes over relatively short durations (typically ≤ 3 months), limiting conclusions about long-term sustainability. Preventive effects remain uncertain. A 66% reduction in knee injury incidence was reported among novice runners using a “land softer” cue; however, this finding came from preventive cohorts, was not derived from runners with established PFP, and may not directly translate to clinical management of symptomatic runners [1]. No direct evidence linking cadence modification to reduced injury rates was identified, reinforcing the need for long-term preventive trials [6]. Partial maintenance of biomechanical and symptomatic gains for up to 6 months is supported by longitudinal studies [2, 8, 23], suggesting potential for sustained adaptation. Still, heterogeneity in protocols limits broader generalisation.

### Implementation and Optimisation Considerations

Narrative and clinical reviews highlight the feasibility of integrating gait retraining tools into routine clinical practice [13, 15]. In addition, feedback-based gait retraining appears most relevant when interpreted as a delivery method to facilitate motor learning and symptom-guided biomechanical adaptation, rather than as an isolated intervention [27]. This interpretation is supported by a critically appraised topic reporting improvements in gait mechanics, pain, and self-reported function in runners with patellofemoral pain following feedback-driven retraining [28]. Accessible tools such as metronomes, mirrors, smartphones, and wearable sensors provide real-time visual or auditory biofeedback, thereby facilitating motor learning. A consistent theme is the relevance of faded feedback, initially intensive and gradually reduced, to promote motor autonomy and long-term retention [14, 15, 25]. This aligns with principles of motor learning and appears essential for transitioning gait modifications from controlled laboratory settings to outdoor running environments.

### Protocol Optimisation and Individualisation

In clinical practice, cadence-based retraining and soft-landing cues may be considered as components of a supervised, symptom-guided rehabilitation plan for runners with PFP, particularly when excessive loading or overstriding is clinically suspected. These strategies should not be interpreted as universally indicated or as substitutes for strengthening, load management, education, and individualised clinical reasoning. Given the heterogeneity of protocols and the predominance of short-term outcomes, clinical decisions should be guided by symptom response, runner preference, training history, and careful progression of running exposure. Optimal retraining protocols vary, but cadence increments of 5–10% are among the most frequently studied approaches and appear feasible when introduced gradually and monitored according to symptom response [1, 6]. Complementary adjustments, including foot-strike modification, increased forward trunk lean, and overstriding reduction, may further reduce variables related to joint loading when applied judiciously [13, 20]. Individual factors such as training history, running experience, anthropometry, and symptom irritability should guide the choice and progression of gait adjustments. Although most evidence focuses on adult recreational runners, groups such as adolescents, older adults, and less active runners remain under-represented, and broader applicability should, therefore, be interpreted cautiously [29].

### Safety and Long-Term Sustainability

Safety outcomes were reported only in some included reviews. Among reviews that described adverse events or tolerability, gait retraining appeared generally well-tolerated when introduced gradually and monitored appropriately; however, reporting was sparse. Reported concerns included transient symptom aggravation or distal calf/Achilles discomfort when biomechanical changes were introduced too rapidly [12, 13]. Medium-term follow-up data suggest that both biomechanical and symptomatic improvements may persist beyond supervised sessions [2, 8]. Nonetheless, the lack of high-quality long-term trials limits definitive conclusions regarding durability. Future studies should include standardised follow-up intervals and assess return-to-running outcomes, training volume progression, and recurrence rates.

### Integration with the Broader Evidence Base

The findings of this umbrella review are broadly compatible with the wider biomechanical and clinical literature suggesting that gait retraining can modulate variables proposed to influence patellofemoral loading, with potential benefits for pain and function in some runners [22, 30]. Nevertheless, pain and function in PFP are influenced by multiple interacting factors beyond joint loading alone, and mechanical changes should not be assumed to translate directly into symptom improvement in every individual. By synthesising higher-quality systematic reviews together with carefully selected narrative evidence, this review supports an individualised, multimodal approach that may incorporate cadence manipulation, foot-strike considerations, and feedback-informed motor learning [20, 21].

### Limitations and Future Directions

Several limitations are inherent to the available evidence. Heterogeneity in intervention protocols, including differences in cadence increments, feedback modalities, training duration, supervision intensity, and comparator conditions, limits comparability across studies. The predominance of short-term outcomes restricts conclusions regarding long-term efficacy and injury prevention. Furthermore, mechanisms underlying clinical improvements, particularly neuromuscular and motor-control adaptations, remain underexplored, and some kinematic variables show mixed or null findings. Although a small number of narrative or clinically appraised reviews were included to address gaps in implementation details, feedback delivery, and mechanistic interpretation not consistently covered by systematic reviews, this choice may have introduced additional risk of bias. To mitigate this, only reviews with explicit methodological transparency and SANRA > = 10/12 were retained, and their findings were used as contextual rather than dominant evidence. Future research should prioritise: (1) standardised retraining protocols with clear progression criteria; (2) identification of responder subgroups based on biomechanics or symptom profiles; (3) integration of gait retraining with strengthening, load management, and education; and (4) long-term trials assessing retention, recurrence, and running exposure metrics.

## Conclusion

This umbrella review suggests that gait retraining may be a feasible adjunctive intervention for runners with patellofemoral pain, particularly when focused on modest cadence increases and/or softer-landing cues within supervised, progressively faded-feedback protocols. The most consistent findings relate to short-term improvements in pain and function and to changes in selected biomechanical variables proposed to influence patellofemoral joint loading. However, heterogeneity across protocols, inclusion of structured narrative evidence, and the predominance of short-term outcomes limit stronger conclusions. Preventive effects remain uncertain, and high-quality controlled longitudinal studies are needed to clarify long-term clinical relevance, optimise protocol parameters, and identify runners most likely to benefit.

## Supplementary Information

Additional file 1.

## Data Availability

All data generated or analysed during this study are included in the published article.
