# Supplementary material for: Gait Retraining for Patellofemoral Pain in Runners: An Umbrella Review of Clinical and Biomechanical Evidence
Source: Sports Med Open. 2026 Jun 15;12:69. doi: 10.1186/s40798-026-01051-8 (PMC13269574; doi:10.1186/s40798-026-01051-8)
Supplement: Supplementary file 1 — Additional file 1. [file 40798_2026_1051_MOESM1_ESM.docx]

# SUPPLEMENTARY MATERIAL

Supplementary file for the manuscript

"Gait retraining for patellofemoral pain in runners: an umbrella review of clinical and biomechanical evidence".

Journal

Sports Medicine - Open

Authors

Ana Carolina Petri; Tamiris Beppler Martins; Taís Beppler Martins; Jaqueline De Souza; Filippo Migliorini; Nicola Maffulli; Rodrigo Okubo

Corresponding author

Prof. Dr. med. Filippo Migliorini, Department of Trauma and Reconstructive Surgery, University Hospital of Halle, Martin-Luther University Halle-Wittenberg, Ernst-Grube-Street 40, 06097 Halle (Saale), Germany. E-mail: [filippo.migliorini@uk-halle.de](mailto:filippo.migliorini@uk-halle.de)

**1. Protocol summary**

Review type: umbrella review of review-level evidence on gait retraining for patellofemoral pain in runners.

Primary population: symptomatic recreational or competitive runners with patellofemoral pain. Broader running populations were considered only when preventive outcomes or mechanistic biomechanical findings relevant to patellofemoral pain were reported separately.

Eligible designs: systematic reviews, meta-analyses, structured narrative reviews, mixed-methods reviews, and clinically appraised reviews focused on gait retraining. Narrative reviews were included only when they reported minimum methodological transparency and achieved SANRA >=10/12.

Outcomes: pain, function, biomechanical variables related to patellofemoral loading, safety/adverse events, and incidence or recurrence of running-related knee symptoms.

Synthesis: structured narrative synthesis by intervention type, comparator when available, outcome domain, and follow-up time point (immediate, short-term >0 to 3 months, mid-term >3 to 6 months). Overlap between included reviews was assessed with an overlap matrix and corrected covered area (CCA).

**2. Database-specific search strategy**

PubMed/MEDLINE:

("running"[Title/Abstract] OR "runners"[Title/Abstract] OR "running-related injuries"[Title/Abstract] OR "patellofemoral pain"[Title/Abstract] OR "PFP"[Title/Abstract] OR "anterior knee pain"[Title/Abstract]) AND ("gait retraining"[Title/Abstract] OR "running technique"[Title/Abstract] OR "running cadence"[Title/Abstract] OR "foot strike"[Title/Abstract] OR "step rate"[Title/Abstract] OR "stride length"[Title/Abstract]) AND ("systematic review"[Publication Type] OR "systematic review"[Title/Abstract] OR "meta-analysis"[Publication Type] OR "meta-analysis"[Title/Abstract] OR "review"[Title/Abstract])

Scopus:

TITLE-ABS ( ("running" OR "runners" OR "running-related injuries" OR "patellofemoral pain" OR "PFP" OR "anterior knee pain") AND ("gait retraining" OR "running technique" OR "running cadence" OR "foot strike" OR "step rate" OR "stride length") AND ("systematic review" OR "meta-analysis" OR "review") )

Manual search: reference lists of included reviews and preprint repositories (medRxiv and SportRxiv).
